# Evolutionary Dynamics of Co-Segregating Gene Clusters Associated with Complex Diseases

**DOI:** 10.1371/journal.pone.0036205

**Published:** 2012-05-14

**Authors:** Christoph Preuss, Mona Riemenschneider, David Wiedmann, Monika Stoll

**Affiliations:** Genetic Epidemiology of Vascular Disorders, Leibniz Institute for Arteriosclerosis Research (LIFA) at the University of Muenster, Muenster, Germany; Biodiversity Insitute of Ontario - University of Guelph, Canada

## Abstract

**Background:**

The distribution of human disease-associated mutations is not random across the human genome. Despite the fact that natural selection continually removes disease-associated mutations, an enrichment of these variants can be observed in regions of low recombination. There are a number of mechanisms by which such a clustering could occur, including genetic perturbations or demographic effects within different populations. Recent genome-wide association studies (GWAS) suggest that single nucleotide polymorphisms (SNPs) associated with complex disease traits are not randomly distributed throughout the genome, but tend to cluster in regions of low recombination.

**Principal Findings:**

Here we investigated whether deleterious mutations have accumulated in regions of low recombination due to the impact of recent positive selection and genetic hitchhiking. Using publicly available data on common complex diseases and population demography, we observed an enrichment of hitchhiked disease associations in conserved gene clusters subject to selection pressure. Evolutionary analysis revealed that these conserved gene clusters arose by multiple concerted rearrangements events across the vertebrate lineage. We observed distinct clustering of disease-associated SNPs in evolutionary rearranged regions of low recombination and high gene density, which harbor genes involved in immunity, that is, the interleukin cluster on 5q31 or *RhoA* on 3p21.

**Conclusions:**

Our results suggest that multiple lineage specific rearrangements led to a physical clustering of functionally related and linked genes exhibiting an enrichment of susceptibility loci for complex traits. This implies that besides recent evolutionary adaptations other evolutionary dynamics have played a role in the formation of linked gene clusters associated with complex disease traits.

## Introduction

Genome-wide association studies (GWAS) have provided proof of principle and revealed numerous disease loci associated with common complex diseases in the human genome. Recent investigations have combined these results with data on genetic variation in human populations, thus linking disease associations with recent evolutionary events. For example, Soranzo et al. [1] showed that a haplotype in a region of long-range linkage disequilibrium (LD), which contains disease loci for coronary artery disease, hypertension and type I diabetes, recently spread by a selective sweep specific to Europeans. Furthermore, evidence from a recent study on deleterious mutations in the human genome has shown that linked deleterious mutations can spread through a population by adaptive selection and cluster in regions of low recombination [2].

Recently, we reported that a region of low recombination on chromosome 5q31 associated with dilated cardiomyopathy, which harbors multiple co-segregating genes, is associated with cardiovascular disease [3]. Evolutionary analysis revealed that the disease-associated genes were clustered along the chromosome in the course of vertebrate evolution as a result of repeated chromosomal rearrangements in different species. Interestingly, these genomic rearrangements coincide with the evolution of heart anatomy in vertebrates, further pointing towards a common pattern of traces of evolutionary forces acting upon the genome and susceptibility to common complex diseases. In the present study we, therefore, investigated whether the clustering of closely linked genes enriched for deleterious mutations is an evolutionary constraint determining complex traits e.g. through clustering of functionally related genes.

While structural and regulatory factors play an important role in the formation of linked gene clusters, the underlying evolutionary dynamics are less understood [4]–[6]. Although recombination continually breaks down genetic associations between weakly related loci, tightly linked gene clusters are a common feature of eukaryotic genomes. For instance, LD in Caucasians [7] contains on average blocks of approx. 60 kb (1–100 kb), but several regions in the genome are characterized by long-range LD (>100 kb). Some well-known examples of such clusters can be found in the human genome including the major histocompatibility complex (MHC) genes on chromosome 6 or the cytokine cluster on chromosome 5q31 with LD extending 500 kb. From an evolutionary point of view, it appears likely that gene order in long-range LD blocks in the human genome did not arise simply by chance but as a result of distinct evolutionary dynamics shaping the genomic architecture. Therefore, we hypothesized that recurrent chromosomal rearrangements in combination with adaptive selection may play a crucial role in the formation of functional gene clusters associated with complex traits.

To test this hypothesis, we performed an analysis of closely linked gene clusters enriched for disease-associated variants in the human genome based on HapMap phase III and 1000 Genomes data [8], [9]. Comparative mapping in multiple vertebrate reference genomes and multiple tests on genetic hitchhiking were used to analyze the evolutionary history of linked disease genes in regions of low recombination. We observed that a large proportion of disease-associated gene clusters in the human genome originates from repeated concerted rearrangements in early vertebrate lineages. Remarkably, these gene clusters overlap with haplotype blocks associated with a range of complex disease phenotypes and show traces of recent selective sweeps.

## Results

### Enrichment of Disease-associated Variants in Regions Showing Low Recombination

In order to test our initial hypothesis that gene clusters in regions of low recombination display an enrichment of disease variants, we screened publicly available data on genotype-phenotype associations throughout the human genome.

Our dataset consisted of a large meta-analysis of Crohn’s disease [10] and two publicly available catalogs of GWAS results recently published by Hindorf et al. and Jonson and O’ Donnell [11], [12]. For our analysis, we classified 18869 disease-associated SNPs across the human genome as those with p-values <1.0×10^–4^ (see also methods section).

We examined genomic regions using a sliding window approach for the 4638 non-overlapping windows of 500 kb. The windows were divided into different bins according to the number of observed disease association per window, estimates of pair-wise linkage disequilibrium and recombination rates which were calculated based on HapMap phase III data for all windows (Materials and Methods).

**Figure 1:**
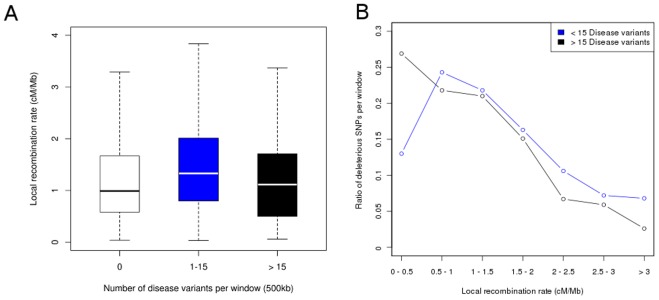
Enrichment of disease variants in regions of low recombination. (**A**) Boxplots displaying local recombination rates for sliding windows of 500 kb harboring a different number of disease variants (0, 1–15,>15). (**B**) Ratio of windows showing an enrichment of disease variants (>15 disease variants) compared to windows without such a clustering (<15 disease variants) for different bins of local recombination rates.

After performing pairwise non parametric test statistics on our dataset, we found a significant difference in recombination rates between windows enriched for more than 15 disease variants compared to windows harboring only a limited number of disease variants (1–15) (Mann-Whitney-Wilcoxon test, P<0.01) (Figure 1A). However, we did not find evidence that windows which show an enrichment of disease variants are over-represented among regions of low recombination. The most pronounced enrichment of disease variants is observed for the bin with the smallest recombination rate (0–0.5 (cm/Mb)) as displayed in Figure 1B when comparing windows harboring more than 15 disease variants to windows containing less disease variants.

Of note, these regions represent a small proportion of the human genome with the top 2.6% (119) of all tested windows showing such enrichment compared to 3553 (76.6%) windows harboring 1 to 15 disease variants. Among the 119 identified regions exhibiting significant clustering of disease variants are several candidate regions which have been independently associated with common complex disease traits, including the MHC region on chromosome 6 and the IBD5 gene cluster on chromosome 5 [11], [12].

In the next step, we were interested if we could find a relationship between the clustering of genes among regions of low recombination and the enrichment of disease variants. Interestingly, while there is no significant correlation in windows with less than 15 disease variants, we found a significant difference in the clustering of 3 or more genes in regions harboring more than 15 disease SNPs as displayed in Figure S1. Also, the gene density differs with an average of 6.77 genes per window in clusters enriched with disease variants compared to 3.44 genes in the remaining windows (Wilcoxon Rank Sum test, P<0.01).

Taken together, these observations support the notion of an enrichment of disease variants in regions of low recombination with a high gene density. When comparing the disease-associated regions we observed distinct differences in clusters of disease SNPs in LD between the different complex traits listed in the GWA dataset. For traits with a strong autoimmune component among the 119 regions enriched for disease variants, such as celiac disease, Crohn’s disease (CD) or childhood asthma, an extensive clustering of SNPs in blocks of long-range LD (>100 kb) was observed, as was for traits that are related to recent adaption to new environments such as skin pigmentation or height. For these traits between 25% and 90% of all disease related SNPs are clustered in blocks of long-range LD. In contrast, common complex diseases including coronary artery disease and bipolar disorder show a clustering of less than 10% of SNPs in long-range LD associated with the disease (Table 1). Table 2 provides an overview of the 32 gene clusters showing the highest enrichment in the top 2.6% regions of low recombination rates (0–0.5 cm/Mb) across the human genome. Among these regions are several loci which have been previously associated with a remarkable disease pleiotropy and enrichment of disease variants including a long-range LD block on chromosome 12q24 harboring variants associated with multiple disease traits [1] and two regions which have been previously associated with inflammatory bowel disease on chromosome 3p21 and 5q31 [13], [14].

**Table 1:** Clustering of selected disease traits in regions of long-range LD among the 119 sliding windows (500 kb) showing an enrichment of disease variants.

Complex Trait	SNPs in LD blocks >100 kb	All GWAS SNPsacross the genome	%	Number of associated regions
Serum uric acid levels	42	44	95	1
Height	70	78	90	4
Childhood asthma	49	58	84	1
Skin pigmentation	63	82	77	1
Celiac disease	18	64	28	4
CD	384	1407	27	36
HDL cholesterol	80	454	18	7
Type II Diabetes Mellitus	377	2345	16	54
Type I Diabetes	159	1158	14	26
Coronary Artery Disease	130	1648	9	21
Hypertension	94	1115	8	19
Bipolar disorder	80	986	8	24
Rheumatoid Arthritis	76	1190	6	20

Legend: Enrichment of disease variants in regions of long-range LD for a number of disease traits obtained from the NIH catalog of GWAS results. The number of LD blocks corresponds to different genomic regions in the genome with pronounced clustering of disease-associated variants.

**Table 2:** Sliding windows encountering the most pronounced clustering of disease variants among regions of low recombination in the human genome.

Chr.	Sliding window start	Sliding window end	Number of disease SNPs	Genefrequency	Associated traits
1	153500001	154000001	36	13	Alzheimer, Crohn’s Disease, Hippocampal atrophy
2	61000001	61500001	27	7	Crohn’s Disease, Psoriasis, Rheumatoid arthritis, Ulcerative colitis
2	123500001	124000001	17	0	Blood Lipids, Type II Diabetes Mellitus
2	198000001	198500001	67	8	Crohn’s Disease
3	48500001	49000001	24	13	Crohn’s Disease
3	49000001	49500001	45	20	Crohn’s Disease
3	49500001	50000001	94	14	Crohn’s Disease
3	50000001	50500001	31	18	Crohn’s Disease
5	40500001	41000001	71	7	Ankylosing spondylitis, Crohn’s Disease
5	99500001	100000001	21	1	Rheumatoid Arthritis
5	129500001	130000001	23	0	Crohn’s Disease, Ischemic stroke
5	130000001	130500001	56	0	Crohn’s Disease
5	130500001	131000001	48	4	Crohn’s Disease
5	131000001	131500001	51	6	Crohn’s Disease
5	131500001	132000001	133	10	Crohn’s Disease
6	26000001	26500001	30	37	Alzheimer’s disease, Bipolar disorder, Height
6	26500001	27000001	21	9	Alzheimer’s disease, Bipolar disorder, Height,
6	27000001	27500001	18	7	Schizophrenia
6	27500001	28000001	23	17	Type I Diabetes
6	28000001	28500001	58	13	Rheumatoid Arthritis, Type I Diabetes
6	28500001	29000001	31	6	Amyotrophic Lateral Sclerosis, Crohn’s disease
6	127000001	127500001	26	1	Crohn’s Disease, Height
7	33000001	33500001	20	0	Crohn’s Disease
10	35000001	35500001	34	4	Crohn’s Disease
10	35500001	36000001	18	4	Crohn’s Disease
12	110500001	111000001	27	8	Celiac disease, Rheumatoid Arthritis, Type I Diabetes
17	35000001	35500001	53	19	Childhood asthma, Crohn’s Disease
17	37500001	38000001	24	16	Crohn’s Disease, Primary biliary cirrhosis
17	41500001	42000001	23	1	Crohn’s Disease
19	42000001	42500001	18	7	Blood Lipids, Crohn’s Disease, Schizophrenia
20	33000001	33500001	52	10	Height, Osteoarthritis
22	28500001	29000001	31	6	Crohn’s Disease

Legend: The 32 windows displaying the highest enrichment of disease variants (>15) among regions of low recombination rates (0–0.5 cM/Mb) across the human genome.

### Genetic Hitchhiking in Regions of Low Recombination and Long-range LD

**Figure 2:**
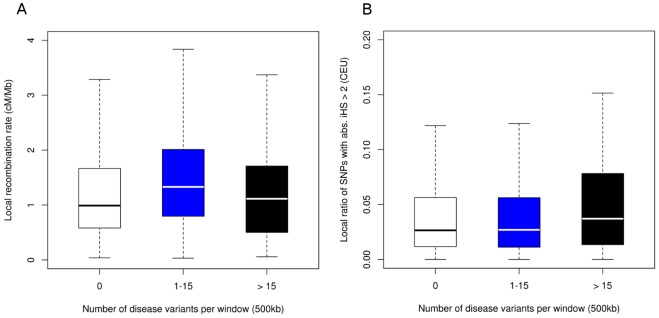
Clustering of iHS signals in regions enriched with disease variants. Boxplots highlighting (**A**) the distribution of mean iHS signals in regions enriched with disease variants (>15) compared to regions with a moderate number of disease associations (1–15) and (**B**) the ratio of strong iHS signals |iHS >2| for these regions.

Several neutral factors, including genetic drift, population size and demographic effects, can generate stretches of low recombination around closely linked loci enriched for disease variants within the human genome. Here, we were primarily interested in the impact of positive natural selection on the observed enrichment of disease variants in regions of low recombination. Therefore, we compared the distribution of signs of positive selection between the bins holding a different number of disease-associated SNPs using the integrated haplotype score developed by Voight et al. [15]. We observed a significant increase in integrated haplotype score (iHS) signals in the top 2.6% of all windows harboring more than 15 SNPs compared to windows without disease variants and windows containing 1 to 15 disease variants (Mann-Whitney-Wilcoxon test, P<0.05) (Figure 2A).

Furthermore, regions enriched for disease variants also display a significant increase in the percentage of strong iHS signals (iHS >2 | iHS <2) compared to the remaining windows with less disease variants as displayed in Figure 2B (Mann-Whitney-Wilcoxon test, P<0.05). Since the iHS statistic has an increased power to detect signals of selection in regions of low recombination, we compared the regions holding a different number of disease associations with matched recombination rates. A small but significant effect was observed for the bin with the lowest recombination rates. This effect could not be observed for regions with higher recombination rates which might be explained by the lack of statistical power to identify strong iHS signals in regions of high recombination (Figure S2).

### Deleterious SNPs Show Population Specific Patterns

**Figure 3:**
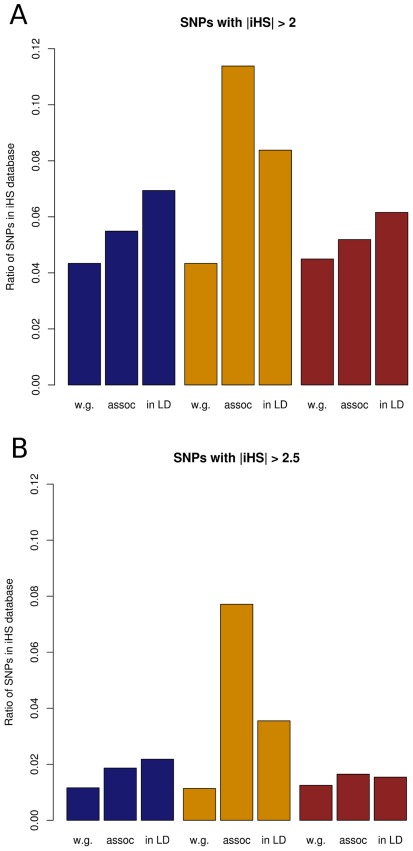
iHS signal percentages for Crohn’s disease in the three HapMap populations. Amounts of (**A**) iHS signals and (**B**) strong iHS signals are given as percentages out of all SNPs genome-wide (w.g.), SNPs associated with Crohn’s disease (assoc) or all SNPs in linkage disequilibrium of associated SNPs at r^2^ > 0.8 (in LD) for the three HapMap populations (Blue: CEU, yellow: ASN, brown: YRI).

To find traces of population specific signs of positive selection, iHS values were retrieved from the Caucasian Europeans/Utah (CEU), East Asians (ASN) and Yoruba/Ibidan (YRI) datasets [15]. iHS signals were retrieved for all SNPs associated with Crohn’s disease (CD) at p<1.0×10^−4^ in the meta-analysis [10] (see methods section). SNPs with |iHS| >2 were defined as “iHS signals” while SNPs with |iHS| >2.5 were defined as “strong iHS signals”. The genome-wide frequency of iHS signals for all SNPs resolves around 4% depending on the population and is lower compared to the disease-associated SNPs in the different populations resolving around 6%-11% as displayed in Figure 3A. The most striking deviation in iHS frequency was observed for the ASN dataset, in which 11.4% of SNPs associated with CD in the European population correspond with iHS signals exhibiting genome-wide significance. In the CEU population, 6.9% of SNPs within these regions fell into this category and 6.2% of the SNPs in the YRI dataset. Figure 3B highlights that when considering only iHS signals with a stringent threshold of >2.5, the same distribution of iHS signals can be observed for the distinct populations. The deviation in iHS signals between the three populations points towards population specific differences that account for the differences in disease allele frequencies between the populations.

**Figure 4:**
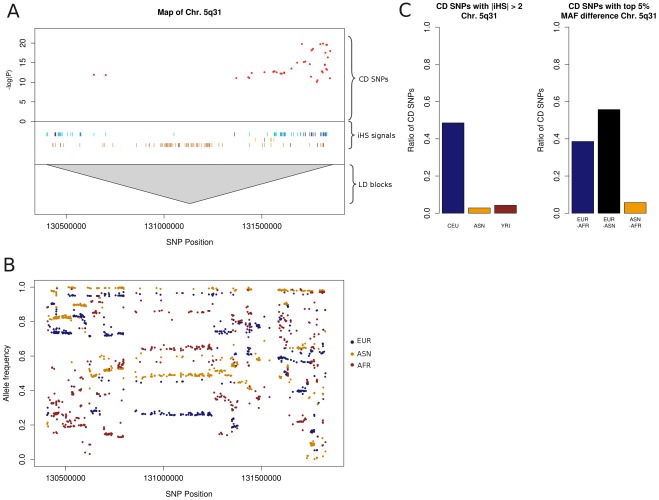
Plots of Crohn’s disease risk locus at chromosome 5q31. (**A**) Map of the 5q31 risk locus containing –log(P) values of SNPs (CD SNPs), LD blocks defined by Proxy SNP with r^2^ >0.8 as well as positions of SNPs considered iHS signals (light colour) or strong iHS signals (darker colour) for the three HapMap populations (blue: CEU, yellow: ASN, brown: YRI). (**B**) Reference allele frequencies of SNPs showing allele frequency differences in the 95^th^ percentile between at least two of three populations according to 1000 Genomes data. (**C**) Percentages of SNPs associated with Crohn’s disease, which are iHS signals (left) or show allele frequency difference in the 95^th^ percentile between populations (right).

In order to test this hypothesis, we used 1000 Genome Pilot 1 data (www.1000genomes.org) and retrieved allele frequencies for the distinct populations. We found signs of population specific differences accompanied by genetic hitchhiking in the two regions exhibiting the most pronounced enrichment of deleterious SNPs. The first locus resides on chromosome 5q31, a region including genes such as *SLC22A4*, *SLC22A5*, *IL3* and *IRF*. The LD block in Figure 4A shows a high iHS signal count in the CEU (8.7% of SNPs) and YRI (7.7% of SNPs) population. Figure 4B displays the selective sweep and the differences in allele frequencies around the IBD5 region, which has been recently associated with genetic hitchhiking in the European population [16]. Among the disease-associated SNPs, 49% in the CEU dataset and only 4% in the YRI dataset display signs of recent selection according to iHS signals (Figure 4C). This reflects differences in the spatial distribution of these signals, as most of the YRI iHS SNPs are clustered in a region without CD association near the gene *FNIP1*, while CEU signals are located in the vicinity of *IL3* and *SLC22A4*. Regarding CD SNP allele frequencies, the European population is very different from the Asian population (56% of SNP with high allele frequency difference) and the African population (39%, see Figure 4C). Consistently, the index SNP risk allele rs12521668-T is very common in Europeans (0.48), while rare in the other populations (ASN: 0.02, YRI: 0.03). The allele rs1050152-T, which is a putative causal mutation in the SLC22A4 gene [17], shows similar allele frequencies (CEU: 0.39, ASN: 0.02, YRI: 0.03). This suggests that selection pressure acted on the European population and favored CD risk alleles. In the Yoruba population, the different environment was accompanied by different selection pressures, which shaped the neighboring *FNIP1* gene. A possible cause of selective sweeps in the 5q31 region might be bacteria, as a recent study has linked this locus to *Mycobacterium tuberculosis* susceptibility [18]. As a study by Huff et al. pointed out that genetic hitchhiking might have also played a role in increasing CD risk by driving alleles of *IRF1* to high frequency while selection pressures were acting on *SLC22A4* due to changes in nutrition [16].

**Figure 5:**
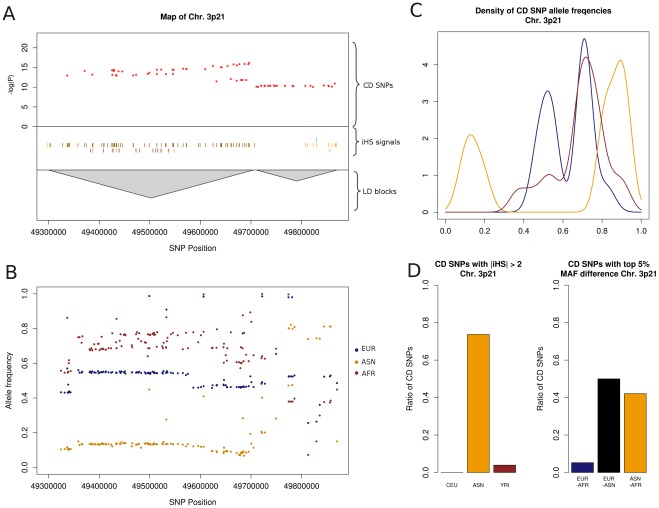
Plots of Crohn’s disease risk locus at chromosome 3p21. (**A**) Map of the 3p21 risk locus containing -log(P) values of SNPs, LD blocks defined by Proxy SNP with r^2^ >0.8 as well as positions of SNPs considered iHS signals (light colour) or strong iHS signals (darker colour) for the three HapMap populations (CEU: blue, ASN: yellow, YRI: brown). (**B**) Reference allele frequencies of SNPs showing allele frequency differences in the 95^th^ percentile between at least two of three populations according to 1000 Genomes data. (**C**) Density plot of reference allele frequencies of SNPs associated with Crohn’s disease. Allele frequencies were retrieved from 1000 Genomes (CEU: blue, ASN: yellow, YRI: brown). (**D**) Percentages of SNPs associated with Crohn’s disease, which are iHS signals (left) or show allele frequency difference in the 95^th^ percentile between populations (right).

The second population specific risk locus is located on chromosome 3p21 and includes genes such as *GPX1*, *MST1* and *BSN* (Figure 5A) [19]. In contrast to the region on chromosome 5q31, the enrichment of disease variants is not accompanied by a selective sweep within the European population. For the *GPX1* gene, a recent selective sweep in the Asian population has already been established [20] and could be reproduced in this study. Within LD of the CD SNPs associated in the European population, a large number of strong iHS signals could be observed in the ASN dataset (72% of SNPs) and a more moderate, but still elevated number in the YRI data (11%, see Figure 5B). Consistently, the allele frequencies show large differences. In the ASN population, most CD SNPs feature extreme reference allele frequencies, which are lower than 0.2 or greater than 0.8 (Figure 5C). These differences in allele frequencies due to recent selection events might have had a profound impact on disease prevalence between populations. While there is a strong association with CD in Europeans, an association signal in individuals of Asian ancestry could not be replicated. The substantial variation in the frequency of disease variants due to recent selective events across human populations may point to differences in disease prevalence between the populations. In the European and African populations, allele frequencies resolve around an intermediate range. This suggests that the strong wide-range selective sweep in the ASN population also had effects on CD SNPs (Figure 5D) and might in fact have lowered the disease risk originating from 3p21. The index SNP rs3197999 was shown to be a non-synonymous coding SNP in the *MST1* gene [21]. Its risk allele A is more common in EUR (0.28) and YRI (0.24) and less frequent in ASN (0.08). Thus, assuming that the risk conferred by disease variants is constant across populations, our data suggest that the common-disease-common-variant hypothesis does not necessarily extend across populations since risk alleles discovered in the European population are found at extremely low or high frequencies in other populations.

### Evolution of Disease-associated Gene Clusters in the Vertebrate Lineage

As outlined before, the most pronounced clustering of disease-associated SNPs is found within larger gene clusters where more than one gene might bare the causal disease association. Among these gene clusters are various regions which have been associated with disease pleiotropy including genes on chromosome 3p21 and 5q31 and 12q21, which have been associated with celiac disease, type 1 diabetes, coronary artery disease (12q24) and osteoarthritis (20q11) [1], [13], [14]. Inspired by our previous study on the co-segregation of cardiomyopathy-associated genes during vertebrate evolution [3], we were interested in the evolutionary dynamics which shaped these gene clusters. Similar to the approach taken previously, we performed comparative genome mapping across six vertebrate genomes in order to trace their emergence throughout vertebrate evolution. Based on the comparative mapping approach we determined shared orthologs between the different evolutionary lineages in mammals, birds, amphibians and fishes. We detected a higher level of conserved gene frequency among the 119 gene clusters in regions of low recombination (Mean = 2.7) compared to the genome-wide average (Mean = 1.6) (Wilcoxon Rank Sum test, P<0.01). We also found an enrichment of conserved gene clusters sharing more than 3 orthologous genes across the lineages within the gene clusters when compared to the genome wide average (Fisher’s exact test, P<0.01). Therefore, we were interested in the formation of the conserved gene clusters sharing a high frequency of orthologs.

Among the 119 regions enriched with disease variants, we could detect 33 distinct, non-overlapping chromosomal regions for which comparative mapping could be performed. For these 33 regions, we identified 16 (48.5%) gene clusters which arose by recurrent chromosomal rearrangement events where the genes disperse in all early vertebrate lineages (birds, amphibians, fishes). 11 (33.3%) gene clusters show species specific rearrangements in at least one branch of the early vertebrate line. 2 gene clusters (6.1%) arose by tandem duplications events and 4 (12.1%) gene clusters share strict synteny between reference genomes. Notably, all gene clusters showing recurrent rearrangements contain putative targets of positive selection in hitchhiked regions. This is in line with a recent observation of a linked gene cluster in butterflies, where a highly polymorphic region played an important role in shaping a region of tightly linked genes associated with a complex adaptive trait [22].

**Figure 6:**
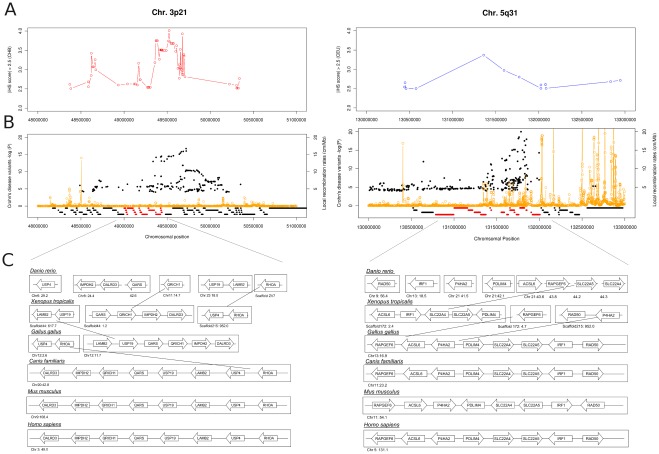
Overview of the co-segregating gene cluster on chromosome 3p21 and 5q31. (**A**) Plot marking the extended haplotype homozygosity for the Asian population (3p21) and the European population (5q31) based on strong iHS signals (iHS >2.5) (**B**) Disease variant distribution (-log (P) values) for the co-segregating gene clusters in the human genome for the region on chromosome 3p21 (48–51 Mb) and 5q31 (130–133 Mb) and the local recombination rates. (**C**) Chromosomal rearrangements and organization of the co-segregating gene clusters in the different vertebrate lineages.

Figure 6 provides a comprehensive view on the various aspects of these regions by displaying (a), traces of selective sweeps (b), the clustering of diseases-associated variants and (c) chromosomal rearrangements in the six vertebrate genomes for two of these regions, namely 3p21 and 5q31. An overview of the remaining clusters not discussed in detail here can be found in the supplementary material (Table S1). The gene cluster on chromosome 3p21 (Figure 6) has been associated with a variety of inflammatory diseases linked to the immune system and inflammation including inflammatory bowel disease and arteriosclerosis. The macrophage stimulating protein *MST1R* and the gluthatione peroxidase 1 (*GPX1)* have both been associated with CD in independent populations and are subject to positive selection in the mammalian lineage. The gene cluster on chromosome 5q31 (Figure 6) harbors several immune related genes, including the interleukins *IL3, IL5* and the interferon regulator *IRF1*, which have been associated with Crohn’s disease in the European population.

The analysis of the HapMap phase III data showed that the LD block harboring the gene clusters is largely intact in all three populations (European [CEU], Chinese [CHB] and African [YRI]). This supports the notion of considerable sharing of haplotypes and inferred recombination points across ethnicities. The orthologous regions show difference in gene order and support the hypothesis that gene rearrangements are evolutionary derived and associated with the locus in the human lineage. Furthermore, the gene clusters are preserved in syntenic blocks on mouse and rat chromosomes, implying that the cluster formation probably took place prior to the divergence of humans and rodents.

## Discussion

To our knowledge, this study represents the first genome-wide evolutionary analysis of disease-associated gene clusters in regions of low recombination in long-range LD across the human genome. Our results show that gene order in long-range LD blocks is highly conserved between mammalian genomes and is, thus, consistent with earlier studies on haplotype structure and gene order in mammals [22], [23]. Our observation of disease-associated gene clusters, however, sparks the question on the evolutionary constraints driving the formation of such clusters prior to the divergence of mammals.

We found that, apart from recurrent tandem duplications, multiple concerted chromosomal rearrangements have played an important role in the formation of at least 16 linked gene clusters in mammalian genomes. Recurrent rearrangements coincide with clustering of previously unrelated genes in early vertebrate species, which decreased the distance between them and thus the likelihood of recombination taking place. When combined, these evolutionary processes may promote the formation of new beneficial allele combinations around positively selected loci resulting in the physical clustering (linkage) of genes along chromosomes. This effect has been recently observed for a polymorphic supergene controlling butterfly mimicry [24].

Chromosomal rearrangements have led to the formation of a long-range LD interval, which is shared between natural populations and which acts as a switch between complex adaptive traits. Allelic combinations that affect wing pattern genes have become locked in the course of evolution. A similar pattern has been observed in sticklebacks where long-range LD can maintain genomic islands of divergence which provides a mechanism for the rapid adaptation to new environments [25].

There is strong evidence suggesting that gene clusters are still under natural selection in the human lineage. We identified several loci in gene clusters whose frequencies vary significantly among human populations due to difference in selection pressures (3p21, 5q31). These gene clusters show strong signatures of positive selection which coincide with an enrichment of more than 15 disease variants around positively selected sites in regions of low recombination.

However, it should be noted that the disease variants might not be the ones under positive selection. If new mutations increase in frequency due to adaptive selection in linked genes, not only the beneficial alleles in the linked genes might hitchhike but also the deleterious mutations. Recent studies revealed a number of regions where genetic hitchhiking resulted in the clustering of disease susceptibility variants nearby unrelated loci [2] [26]. Furthermore, the weakly selected variants in regions of low recombination might be explained by the Hill-Robertson (HR) effect [27]. Many of the disease variants detected within this study are probably so weakly selected that Hill–Robertson interference undermines the effective strength of selection upon them, when recombination is rare. Where local recombination rates are reduced by the effects of recent selective sweeps, there will be a smaller amount of polymorphism and hence lower divergence [28]–[30]. On the population level, divergence and F_ST_ values are expected to be higher for genes or genomic regions for which recombination rates are low [31]. Also there is evidence for this in human populations [32] and in nematodes [33].

One striking observation in our study is the relationship between allele frequency spectra, clustering of disease variants and the effects of recent selective sweeps for different pairs of human populations. While all disease associations originate from individuals with European ancestry, the most striking signs of positive selection, according to iHS statistics, are found among the Asian population suggesting that selection has acted in different ways and timescales on the adherent populations.

Since the populations have diverged, Europeans and Asians have encountered a different population history with smaller effective population sizes. Therefore, natural selection might be not very efficient in reducing weak deleterious mutations in these populations, but in turn lead to their increase in frequency due to the effect of background selection [34]. Furthermore, the population specific patterns in regions enriched for disease variants can be explained by selective sweeps that are shared across populations. Such global selective sweeps might arise when one allele that increased in frequency due to positive selection in one population enters another population through the process of migration and reduce variation at linked sites. Since Europeans and Asians exchanged genes more recently between each other compared to Africans, more shared selective events should be expected between these two populations [32], [35]. Also there are various other scenarios which could explain the divergence between observed disease associations and selective sweeps between populations. Disease prevalence between populations may differ and the associated risk variants can have different effect sizes so that variation across populations can exist in the underlying determinants of the same disease. The example from the *APOE* variants associated with Alzheimer shows that risk variants for one population are most likely to be determined within the same population rather than within samples from different populations [36].

Finally, the absence of natural selection in the European population might have had an impact on disease prevalence within the population. Selection is thought to have optimized immune function with respect to expected longevity and the impact of various pathogen interactions throughout lifetime. The increase of the expected longevity and the reduced load of pathogen interactions lead to the possibility of an accumulation of oxidative damage throughout the lifetime of an individual. Inflammatory disease, with its shift towards chronic inflammation, might be the result of the immune system that provides effective protection in early life when natural selection is almost blind to inflammatory diseases that arise after the reproductive phase. This might be relevant for the region on chromosome 3p21, where several selenoproteins (*GPX1)* and other immune genes are located that are involved in the protection and regulation of the oxidative stress response. These genes involved in the antioxidant defense have been shown to play a role in inflammatory bowel disease [37]. A limitation of this study is the genetic map available and the outlier statistics used to detect recent selection events. For our study, we used a pedigree-derived human genetic map that is based on the direct observation of recombination events. However, genetic maps that are based on patterns of LD in a population are sensitive to the increase in LD due to natural selection [38], [39].

In future studies of these phenomena, a finer-scale map should allow a better characterization of the relationship between population differentiation and recombination rate and improved statistical power in capturing its causes. As recombination rates vary between populations [40] and background selection may occur at different genetic distance scale than hitchhiking it remains a challenging task to detect the traces of recent selection that led to the clustering of disease variants in regions of low recombination.

## Materials and Methods

### Genome Clustering for Disease-associated SNPs

In order to identify genomic regions with exceptionally high rates of disease SNPs, data on genetic variants associated with common complex diseases was obtained from the NIH catalog of GWA studies (www.genome.gov/gwastudies) accessed in June 2011, the publicly available catalog of GWA results and from the International Inflammatory Bowel Disease Genetics Consortium homepage [10]–[12]. Associated variants were included if they met the statistical significance (SNP-trait p-value <1.0×10^−4^) in the overall (initial GWAS + replication) population and can be found in Table S2. For each SNP that met these criteria, SNP-phenotype information, chromosomal region (from ENSEMBL); gene (as reported); rs number and risk allele (as reported) were collected. Duplicated entries between data sets have been removed based on rs accession identifiers and chromosomal locations. Windows that were less than 10 Mb apart from the centromers and telomers and 500 kb windows for which recombination rates could not be obtained were removed from the analysis. The remaining 4638 windows were divided in equally sized bins by their recombination rates and number of disease variants.

The density of SNPs was measured as a function of local recombination rate using CEU, CHB and YRI SNPs from the HapMap phase III project. Assuming that the rate of disease SNPs is constant across the genome, a Poisson distribution was used to evaluate the excess number of deleterious SNPs in each of the 4638 sliding, non overlapping 500 kb windows.

### Estimation of LD and Mining Regions of Low Recombination in the Human Genome

Linkage disequilibrium in the human genome on disease variants was calculated based on 1000 Genome Pilot 1 data (www.1000genomes.org). Genetic data for all four HapMap populations was downloaded and analyzed for regions of linkage disequilibrium using the Haploview software tool [41].

A sliding window approach was used to compute r-square, a common measure of linkage disequilibrium, between each pair of disease SNPs that are located within a non overlapping window of 500 kb with a threshold of r^2^ >0.8. Haploview computes 95% confidence intervals (CI) for using the uniform distribution. Next, the algorithm by Gabriel and colleagues [42] implemented in Haploview was used to define extended linkage disequilibrium in the regions harboring more than two disease SNPs exceeding a maximum LD distance greater than 100 kb. LD blocks containing at least two disease SNPs in all four HapMap populations were selected for further evolutionary analyses. The annotation of genes within these regions of extended LD was performed using the ENSEMBL database version 61 and the biomart interface in the Bioconductor software suite (www.bioconductor.org) [43].

### Detection of Genomic Rearrangements and Levels of Ortholog Gene Frequencies Among Conserved Gene Clusters in the Human Genome

The evolutionary history of LD blocks in the human genome was assessed by using a comparative analysis between six vertebrate reference genomes, including mammals (*Homo sapiens, Mus musculus, Canis familiaris*), birds (*Gallus gallus*), amphibians (*Xenopus tropicalis*) and fishes *(Danio rerio).* Genes only annotated in human were excluded from the analysis, because the absence of these genes in other vertebrate organisms is mostly due to limitations in annotation of less well characterized genomes.

Orthologs for the human genes were obtained from ENSEMBL compara release 48 for the six species [44]. The analysis of conserved genes in ortholog species was performed using the R package ‘biomaRt’ from the Bioconductor software suite [41]. The database ‘ensemble’ - Ensembl BioMart database - and the dataset ‘hsapiens_gene_ensembl’ was used to retrieve ortholog information for the six vertebrate reference species using a specific filter to retrieve ortholog gene annotations. Attributes of distinct information about gene id, chromosome name, start and end position, etc. was queried. With a window sliding approach (sliding window size of 500 kb) the number of conserved genes per window and chromosome were calculated for each reference species.

### Signs of Recent Positive Selection and Genetic Hitchhiking in Regions of Low Recombination Across the Human Genome

Hitchhiking regions were defined by genomic windows that were identified by two or more out of 4 tests for hitchhiking [45]. We screened for evidence of positive selection and selective sweeps in regions of low recombination using the publicly available data from the Haplotter tool provided by Voight et al. (http://haplotter.uchicago.edu/) [15] and the SNP@Evolution database (http://bighapmap.big.ac.cn/) [46]. We captured multiple signals of positive selection across conserved hitchhiked regions including empirical values of heterozygosity (HET), F_ST_ values, iHS signals and Fay and Wu’s statistics for each associated genomic window. Furthermore, two recent studies on recent positive selection of deleterious alleles were screened in order to detect overlaps between regions showing evidence for genetic hitchhiking [2], [47].

### Population Specific Differences and Allele Frequency Deviations

Crohn’s disease association data was acquired from the International Inflammatory Bowel Disease Genetics Consortium homepage (http://www.ibdgenetics.org/downloads.html). This dataset originates from a meta-analysis of six genome-wide association studies with individuals of European ancestry [10]. Within Crohn’s disease susceptibility loci 3p21 and 5q31, all SNPs associated at p-value <1.0×10^−4^ were selected for further analysis. For these SNPs, regions of high linkage disequilibrium were defined by identifying proxy SNPs via the proxy search function of SNAP [48], using the CEU panel of the 1000 Genomes Pilot 1 dataset and a threshold of r^2^ >0.8. These regions were examined for signals of recent positive selection and population-specific allele frequencies. As a measure of recent selection integrated haplotype score (iHS) data [15] was downloaded from Haplotter (http://haplotter.uchicago.edu/) for the populations Caucasian Europeans in Utah (CEU), East Asians (ASN) and Yoruba from Ibidan (YRI). For each of the populations, values of |iHS| >2 approximately reflect the extreme 4% of signals and were considered “iHS signals”. Positive and negative iHS values were treated equally, as no discrimination between ancestral and derived alleles was needed. SNP allele frequencies for European, East Asian and African populations were determined from 1000 Genomes data [8].

## Supporting Information

Figure S1 **Recombination rates for gene clusters harboring a different number of disease-associated variants.** Relationship between local recombination rates and the physical clustering of genes for sliding windows (500 kb), harboring a different number of disease-associated variants. A significant difference in local recombination rates was only observed for gene clusters showing an enrichment of more than 15 disease variants (Wilcoxcon Rank Sum test, P<0.01).
(TIFF)

Figure S2 **Differences in iHS scores for regions with matched recombination rates.** Matched recombination rates displaying the differences in iHS signals for the European population between windows enriched for disease associations (>15 disease variants), windows harbouring only a limited number of disease variants (1–15) and windows showing no signs of disease associations. Significant difference could be observed for the bin with local recombination rates between 0.5–1.0 for the three groups (Kruskal Wallis test on bin affiliation: χ^2^ (2) = 7.89, P<0.05 [p = 0.019]). For the remaining bins, only a trend towards higher iHS signals could be observed for the windows showing an enrichment of disease variants. This is due to the low sample sizes affecting the distribution of iHS signals in regions of high recombination rates.
(TIFF)

Table S1 Overview of the 33 regions for which comparative evolutionary analysis was performed. For each region, number of disease-associated variants according to genotype-phenotype classification, number of genes, HGNC identifiers and complex phenotype associations are shown. In addition, recently reported targets of positive selection with immune related functions are highlighted and the individual populations for which strong signs of recent selection (iHS>2.5) could be observed.
(XLSX)

Table S2 Catalog containing genotype-phenotype associations. Dataset on the 18869 genotype-phenotype associations obtained from the NIH catalog of GWA studies (www.genome.gov/gwastudies) accessed in June 2011, the publicly available catalog of GWA results [10] and from the International Inflammatory Bowel Disease Genetics Consortium homepage (http://www.ibdgenetics.org). Associated variants were included if they met the statistical significance (SNP-trait p-value <1.0×10^–4^) in the overall (initial GWAS + replication) population. For each SNP that met these criteria, SNP-phenotype information, chromosomal position (from ENSEMBL 61); rs number and the disease catalog are listed.
(TXT)
