# Supplementary figures and images for: Evolutionary Dynamics of Co-Segregating Gene Clusters Associated with Complex Diseases
Source: PLoS One. 2012 May 14;7(5):e36205. doi: 10.1371/journal.pone.0036205 (PMC3351447; doi:10.1371/journal.pone.0036205)

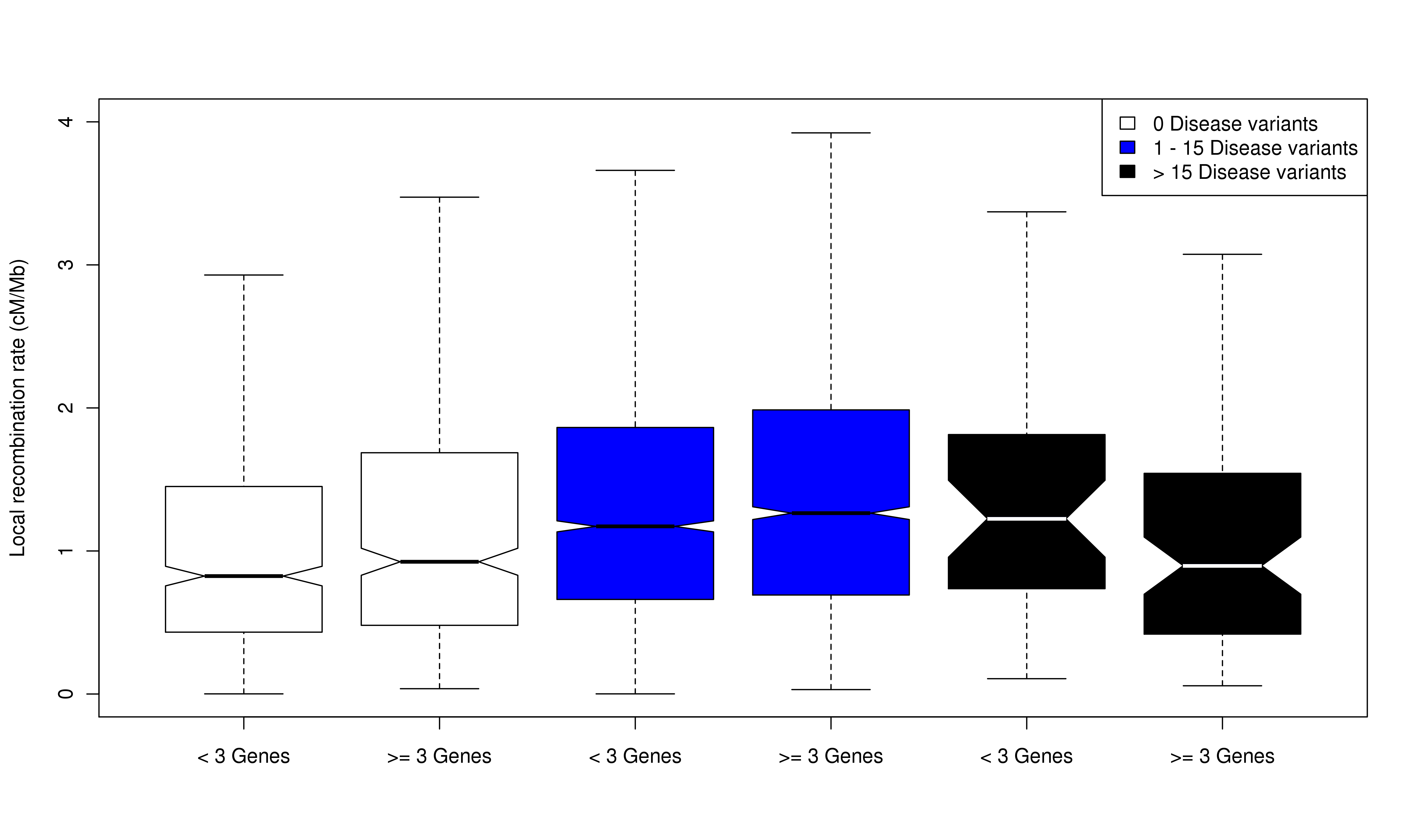

Supplement: Figure S1 — Recombination rates for gene clusters harboring a different number of disease-associated variants. Relationship between local recombination rates and the physical clustering of genes for sliding windows (500 kb), harboring a different number of disease-associated variants. A significant difference in local recombination rates was only observed for gene clusters showing an enrichment of more than 15 disease variants (Wilcoxcon Rank Sum test, P<0.01). (TIFF) [file pone.0036205.s001.tiff]

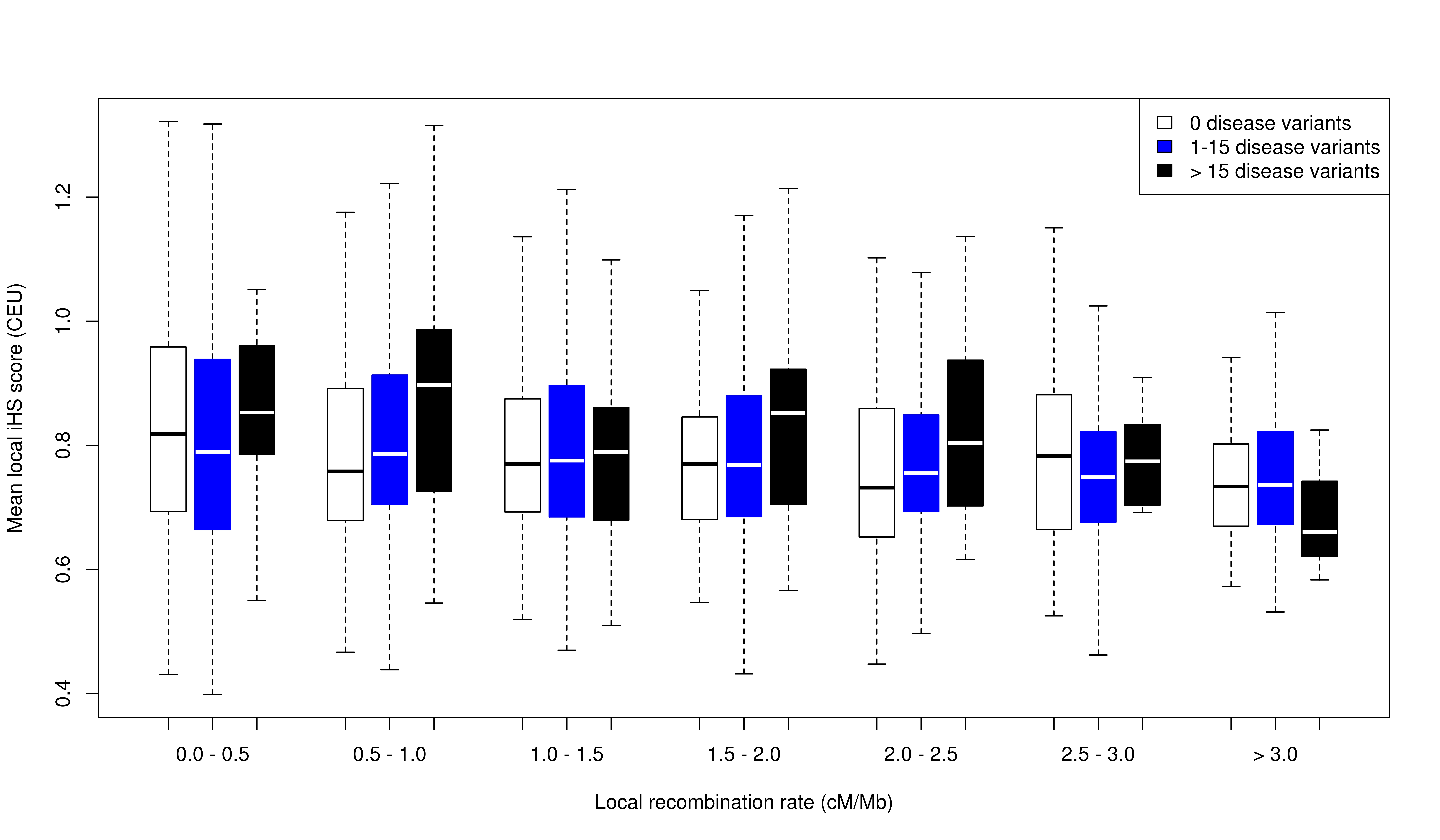

Supplement: Figure S2 — Differences in iHS scores for regions with matched recombination rates. Matched recombination rates displaying the differences in iHS signals for the European population between windows enriched for disease associations (>15 disease variants), windows harbouring only a limited number of disease variants (1–15) and windows showing no signs of disease associations. Significant difference could be observed for the bin with local recombination rates between 0.5–1.0 for the three groups (Kruskal Wallis test on bin affiliation: χ2 (2) = 7.89, P<0.05 [p = 0.019]). For the remaining bins, only a trend towards higher iHS signals could be observed for the windows showing an enrichment of disease variants. This is due to the low sample sizes affecting the distribution of iHS signals in regions of high recombination rates. (TIFF) [file pone.0036205.s002.tiff]
